# Formulation and evaluation of alternative to beeswax for vegan lipsticks

**DOI:** 10.1111/ics.13060

**Published:** 2025-03-12

**Authors:** Manuela Loiacono, Luigi Padovano, Miryam Chiara Malacarne, Simone Conti, Enrico Caruso

**Affiliations:** ^1^ Roelmi HPC s.r.l. Origgio VA Italy; ^2^ Department of Biotechnology and Life Sciences (DBSV) University of Insubria Varese VA Italy

**Keywords:** beeswax, lipsticks, mimic beeswax, natural wax, vegan cosmetics

## Abstract

Honeybees' success is strictly linked to the chemical and application properties of their products: honey, beeswax (BW), venom, propolis, pollen and royal jelly. Among these products, BW, a natural compound secreted by bees, is particularly valued for its stability and is widely used in cosmetics for make‐up and skincare production or in dermatology to produce creams. In recent years, there has been a growing awareness of the critical role these insects play in the ecosystem. Animal‐derived ingredients are controversial and borderline with consumers' necessities. Therefore, whenever possible, similar ingredients from other sources are sought. The demand for vegan products is a global trend that influences all segments of consumer behaviour, including the choice of cosmetics. Given the growing consumer demand for eco‐friendly products, BW, commonly used in lipstick formulations, needs to be replaced with a vegan alternative. In this paper, we report the development of a completely vegan lipstick. To predict the behaviour of structuring wax in a complex system, a compatibility study of the ABWAX® MIMIC BEESWAX MK, a vegan beeswax alternative (vBWA), with oils and colours was performed. The ABWAX® MIMIC BEESWAX MK and ABWAX® WHITE BEESWAX F.U. demonstrated similar thermal characteristics and penetration curves, showing overall comparable performances in finished products. We can conclude that ABWAX® MIMIC BEESWAX MK could be an innovative and highly effective alternative to animal‐derived waxes in cosmetics.

## Graphical abstract

Characterization of a cosmetic ingredient as a vegan alternative to beeswax. Investigation of binary oil–wax systems for cosmetic applications. Development of a predictive strategy for the formulation of vegan stick cosmetics.

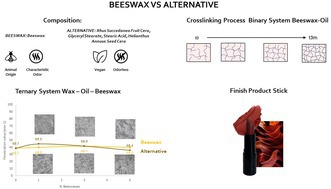

## Résumé

L’essor des abeilles mellifères est étroitement lié aux propriétés chimiques et d’application de leurs produits : le miel, la cire d’abeille, le venin, la propolis, le pollen et la gelée royale. Parmi ces produits, la cire d’abeille, un composé naturel sécrété par les abeilles, est particulièrement appréciée pour sa stabilité et est largement utilisée en cosmétique pour la production de maquillage et de soins de la peau ou en dermatologie pour la production de crèmes. Ces dernières années, le rôle essentiel que jouent ces insectes dans l’écosystème a fait l’objet d’une prise de conscience croissante. Les ingrédients d’origine animale sont controversés et se trouvent dans la zone grise des besoins des consommateurs. C’est pourquoi, dans la mesure du possible, des ingrédients similaires provenant d’autres sources sont recherchés. La demande de produits végans est une tendance mondiale qui influence tous les segments du comportement des consommateurs, y compris le choix des produits cosmétiques. Compte tenu de la demande croissante des consommateurs pour des produits respectueux de l’environnement, la cire d’abeille, couramment utilisée dans les formulations de rouges à lèvres, doit être remplacée par une alternative végane. Dans cet article, nous présentons le développement d’un rouge à lèvres entièrement végan. Pour prédire le comportement de la cire structurante dans un système complexe, une étude de compatibilité d’ABWAX® MIMIC BEESWAX MK, une alternative végane à la cire d’abeille (vegan beeswax alternative, vBWA), à base d’huiles et de colorants, a été réalisée. Les produits ABWAX® MIMIC BEESWAX MK et ABWAX® WHITE BEESWAX F.U. ont démontré des caractéristiques thermiques et des courbes de pénétration similaires, affichant des performances globales comparables dans les produits finis. Nous pouvons conclure que le produit ABWAX® MIMIC BEESWAX MK pourrait être une alternative innovante et très efficace aux cires d’origine animale dans les produits cosmétiques.

## INTRODUCTION

In the cosmetics industry, active ingredients, functional substances and additives can originate from both chemical and animal sources [1]. Animal‐derived products are often multifunctional, containing a variety of substances with different functions and cosmetic uses [2]. The main animal‐derived ingredients come from insects, mainly bees; land animals, mainly snails [3]; and marine animals [4].

Over the last decade, there has been a shift in consumer preferences towards natural cosmetics [5] free of synthetic chemicals [6, 7, 8, 9]. Sustainability is now a key concern for cosmetic ingredients among informed and vigilant consumers [10]. Unfortunately, however, animal‐derived ingredients are also a controversial issue in the cosmetics industry due to ethical and ecological reasons [11, 12, 13]. As a result, growing consumer demand for eco‐friendly products has driven the market towards more vegan products [14, 15] and industries to increasingly use ingredients from other sources whenever possible [16, 17].

The term “vegan” now represents not only attention to the origin of cosmetic ingredients but also a broader lifestyle philosophy applied to skincare. According to some reports, the global vegan cosmetics market is expected to reach a value of 20.8 billion dollars by 2025 [18]. Vegan cosmetics are not limited to natural or organic ingredients; as long as they are not of animal origin, they also involve green chemistry ingredients to create environmentally friendly formulas that reduce the use of polluting substances [19, 20].

In the cosmetics sector, one of the most widely consumed products is lipsticks, which offer a wide range of colour shades and textures to satisfy the growing demand [21, 22, 23]. A key component in lipstick formulation is wax, which is crucial for achieving an excellent final product [24, 25, 26]. Waxes are used as structuring agents in lipsticks because their lattice provides stability [27].

Among animal waxes, beeswax (BW), produced by bees of the genus Apis (*A. mellifera* and *A. cerana*) [28], is recognized as an essential ingredient. BW is secreted by worker bees' wax glands [29, 30]. Initially liquid when just secreted, BW solidifies upon contact with air and changes colour from white to yellow due to contact with honey and pollen [31]. It is used as a glazing agent for enhancing glossiness and to structure solid cosmetics products [10, 19, 30, 32]. Additionally, BW contains small amounts of natural antibacterial agents that help soothe the pain associated with infections [33, 34].

Animal‐derived ingredients are a controversial; as a result, industries are increasingly using similar ingredients from other sources whenever possible.

From a vegan perspective, waxes must be derived from plants [35], minerals, or synthetic sources made of esters of long‐chain fatty acids. Generally, plant waxes contain very long‐chain fatty acids, alcohols, ketones and sterols. All the aliphatic components of plant waxes are synthesized from saturated long‐chain fatty acids [25].

Vegetable waxes can be extracted from various parts of the plants, such as leaves (carnauba and candelilla wax from *Copernicia pruniera* and *Euphrobia antisyphilitica*, respectively) [36, 37], flowers (sunflower wax, SFW, from *Helianthus annuus*) [38, 39], fruits (berry wax from *Myrica cordifolia*), hull (rice bran wax from *Oryza sativa*), or seed, as in the case of jojoba (*Simmondsia chinensis*).

As future cosmetics need to be increasingly sustainable and “green” [40, 41, 42, 43], this research paper focuses on the development of a vegan eco‐friendly lipstick. We formulated the stick using a vegan wax, ABWAX® MIMIC BEESWAX MK. Compatibility studies with commonly used cosmetic oils and colours allowed us to predict the performance of ABWAX® MIMIC BEESWAX MK in complex systems. A comparative study resulted in a vegan stick formulation with performance comparable to traditional BW‐containing lipsticks.

## EXPERIMENTAL SECTION

### Materials

Table 1 shows all the materials used, showing their function and chemical class.

**TABLE 1:** Structuring ingredients and oils used in tests.

	Trade name	INCI	NOI (ISO16128)	Functions	Chemical class
Structuring ingredients	ABWAX® WHITE BEESWAX F.U.	*Beeswax*.	1	Plasticizer, structuring agent	Wax
	ABWAX® MIMIC BEESWAX MK	*Rhus Succedanea Fruit Cera, Glyceryl Stearate, Stearic acid, Helianthus Annuus Seed Cera*	1	Plasticizer, structuring agent	Wax
	ABWAX® SUNFLOWER WAX PEARLS	*Helianthus Annuus Seed Cera*	1	Plasticizer, structuring agent	Wax
Oils	Vaseline	*Petrolatum*	0	Emollient	Hydrocarbon
	Octyldodecanol	*Octyldodecanol*	1	Emollient	Alcohol
	Emotion® air	*Ethylhexyl pelargonate*	0.6	Emollient	Monoester
	Emotion® skin	*Triolein, Glyceryl Dioleate*	1	Emollient	Diester
	MCT	*Caprylic/Capric Triglyceride*	1	Emollient	Triester
	Castor oil	*Ricinus communis seed oil*	1	Emollient	Natural oil
Ingredients of finished products	ColorGlam™ Light RedOx	*CI 77491, C8‐12 Acid Triglyceride*		Coated iron oxide	Colouring
	AmiPearl® Earth 1806 Chestnut Brown	*Mica, CI 77891, Tin oxide*		Inorganic pigment	Texturizer

### Procedure

#### Raw materials analysis

ABWAX® WHITE BEESWAX F.U. and ABWAX® MIMIC BEESWAX MK were characterized using analytical methods described in the European Pharmacopoeia monograph for white BW, including chemical analysis like acid value, saponification value, iodine number and ester value and thermal analysis like differential scanning calorimetry (DSC), congealing point (ASTM D938) and drop point (DP) [44]. Thermal analysis was conducted using two instruments from Mettler Toledo (Mettler‐Toledo S.p.A., Milan, Italy): a STARe System DSC 3 for Differential Scanning Calorimetry (DSC) and a Dropping Point System DP70 for Drop Point (DP) measurements.

#### Microscope study of oil–wax crosslinking

The binary oil–wax systems were prepared by melting the wax and oil together at a temperature above the waxes melting point (MP). The wax w/w% in the oil were 1%, 3%, 5% and 10%. The oils tested were chosen based on their different characteristics: Petrolatum is a non‐polar hydrocarbon, Octyldodecanol is a polar alcohol oil, Caprylyl/Capric Triglyceride is a polar triester, and Castor oil is a viscous polar oil. The mixtures were stirred continuously while melting to ensure homogeneity.

A drop of each mixture was placed on a preheated microscope slide. The slides were heated to a temperature above the MP of the wax to ensure that the wax was completely melted and evenly distributed. A coverslip was placed on each drop to prevent contamination, securing the sample to the microscope slide.

Samples were then analysed using an Olympus IX81 microscope (Olympus LS, Tokyo, Japan) equipped with an Optika C‐P20Cm digital camera (Optica Italia, Ponteranica, Italy). The microscope was set to a magnification of 40X, and an image of each sample was captured at *t*_0_ (immediately after melting the wax). The samples were then analysed again after 1 h, 7 days, 1 month and 3 months.

#### Oil–wax synergies study

The experimental procedure began by preparing a wax–oil ternary system composed of BW or vBWA, SFW and oil. Samples made of BW and its vBWA were prepared at 1%, 3% and 5% concentration with 24%, 22% and 20% SFW and 75% of each oil. These synergies were melted under stirring to ensure homogeneity and slowly cooled to RT. To evaluate their resistance to penetration and hardness, the ternary synergies were first heated to their melting point (MP) with continuous stirring. The samples were then slowly cooled to 25°C and subsequently analysed using a manual penetrometer (650/SEM748, Montepaone S.R.L., San Mauro Torinese, Italy). The same samples were also analysed using a DP and optical microscope, as reported in Section 2.2.2.

#### Lipstick formulation—production method

Each ternary synergy was heated until 85°C and completely melted. The molten synergy was poured into a metal mould for lip balms, which was preheated to 85°C and lubricated with a food‐grade silicone spray to prevent thermal shock and for easy removal. The mould was maintained at room temperature for 15 min, followed by chilling at −18°C for another 15 min to ensure complete solidification. Once the consistency allowed handling, the mould was disassembled and the formed sticks were packaged [45]. The obtained lip balms were evaluated through a sensory panel test to assess the performance and consumer perception of the product. A group of 25 trained panellists was involved in the descriptive quantitative analysis (QDA) to evaluate the stick smoothness and spreadability by assigning scores ranging from 1 (low) to 10 (high). This data allowed for the differentiation between the creamy and control sticks. Spreadability was further categorized as low, medium and high. The stability of the sticks was monitored over time by storing them at 25°C and 40°C for 1, 3 and 6 months.

#### Development of finished products

The raw materials were weighed in a beaker. Then the mixture was heated until completely melted under continuous stirring. Once the temperature reached 85–90°C, the mixture was homogenized for 30 s at 10 000 rpm with an OV5 dispersion homogenizer (Velp Scientifica, Usmate, Italy). The homogenized mixture was heated again to 85°C and then poured into a metal lipstick mould. The formulation steps follow those described in Section 2.2.4.

To assess the product's sensory characteristics and consumer perception, expert panellists were recruited for a sensory evaluation (as described in Section 2.2.4). They were asked to evaluate key attributes like spreadability, glossiness, greasiness, ease of application (not messy) and colour payoff [46]. The lipsticks were subjected to a stability test, and monitored for up to 6 months at storage temperatures of 25°C and 40°C.

## RESULTS AND DISCUSSION

### Raw materials analysis

The chemical–physical parameters, such as acidity, saponification, iodine number and thermal analyses of the BW, were analysed using an internationally recognized method, namely the European Pharmacopoeia's monograph on beeswax. Similarly, the same monograph was used for the analysis of the vBWA, to obtain comparable values. As can be seen from Table 2, the results of the vBWA fall within the ranges dictated by the European Pharmacopoeia's monograph [44]. The DSC analysis was carried out to compare the thermal transitions of ABWAX® WHITE BEESWAX F.U. and ABWAX® MIMIC BEESWAX MK.

**TABLE 2:** Physical–chemical characteristics of ABWAX® WHITE BEESWAX F.U. and ABWAX® MIMIC BEESWAX MK.

Parameter	Requirement		Methods
	ABWAX® WHITE BEESWAX F.U.	ABWAX® MIMIC BEESWAX MK	
Composition	*Beeswax*	*Rhus Succedanea Fruit Cera, Glyceryl Stearate, Stearic Acid, Helianthus Annuus Seed Cera*	‐
Acid value	17–24	17–33	As per «Beeswax, white» Ph. Eur. Monograph
Saponification value	87–104	87–120	As per «Beeswax, white» Ph. Eur. Monograph
Iodine number	≤6	≤ 6	As per «Beeswax, white» Ph. Eur. Monograph
Ester value	70–80	70–87	Saponification value ‐ Acid value
MP^a^	64–70	64–70	DSC
DP^b^	61–66	64–70	Ph.Eur.2.2.17 (Method B)
Congealing Point	60–67	60–67	ASTM D938

^a^ The MP was obtained from the DSC thermic profile.

^b^ The DP analysis was repeated 5 times to establish a temperature range.

**FIGURE 1:**
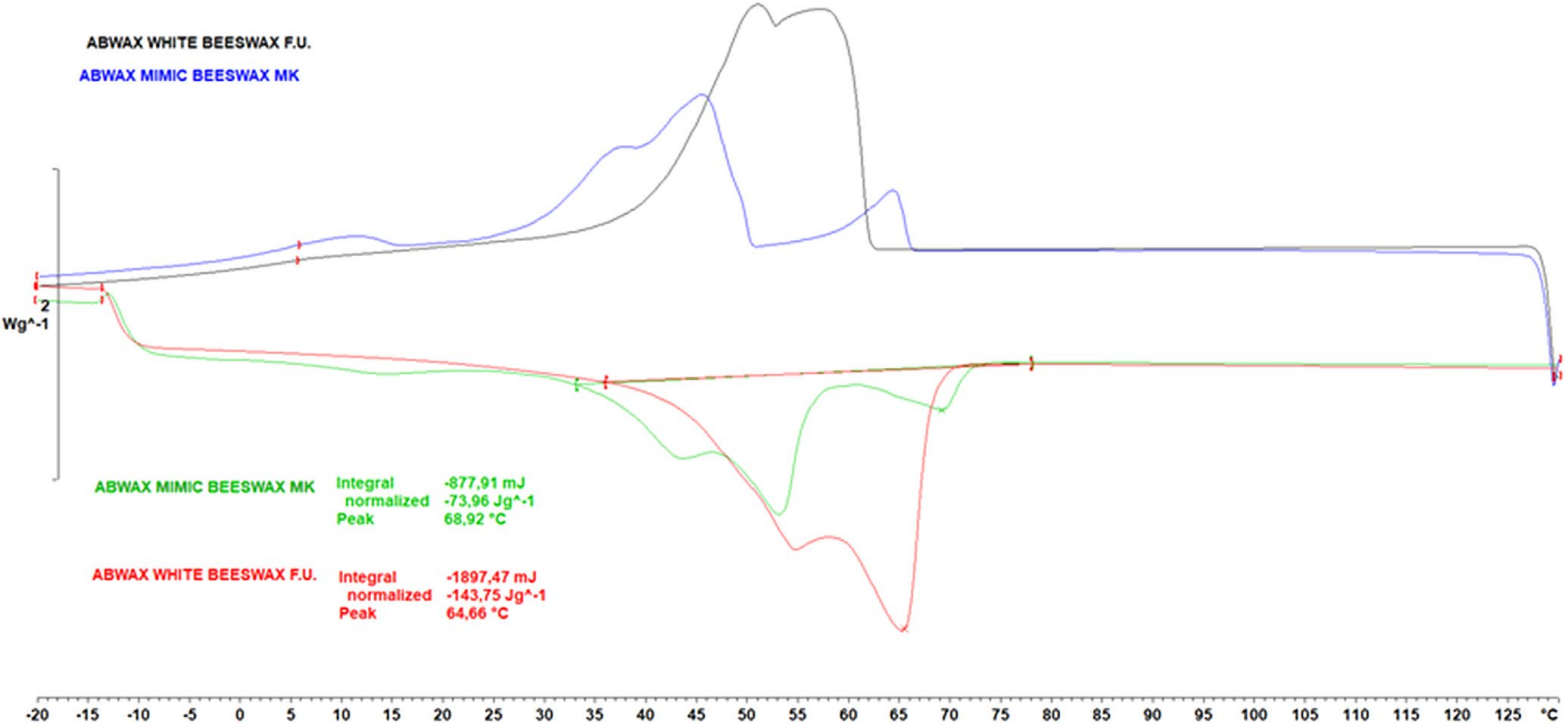
DSC curve ABWAX® WHITE BEESWAX F.U. and ABWAX® MIMIC BEESWAX MK Cooling and melting profile curves of ABWAX® WHITE BEESWAX F.U. (Cooling: Black, Melting: Red) and ABWAX® MIMIC BEESWAX MK (Cooling: Blue, Melting: Green).

An examination of DSC analysis of ABWAX® WHITE BEESWAX F.U. and ABWAX® MIMIC BEESWAX MK shown in Figure 1 provides valuable insights into the nature of phase transitions and thermal properties of the materials [39].

The results revealed that both materials exhibit similar MPs, with BW having an MP of 68.92°C and the alternative having an MP of 64.66°C. This close resemblance in MPs suggests a convergence in thermal behaviour despite the differences in the chemical composition of the two materials.

Based on the results obtained from the thermal and chemical characterization, it is reasonable to expect that their molecular weights are also comparable.

As shown in Figure 1, another significant measure obtained from DSC analysis is the final melting temperature (*T*_fmelting_), which represents the final temperature of the melting process. In our study, BW exhibited a *T*_fmelting_ of 70°C, slightly lower than the 72°C observed for the vBWA. However, it is important to note that this difference is minimal and may not significantly impact the performance of formulations containing these materials.

The vBWA curve shows multiple peaks that suggest the possibility of a more complex composition compared to BW, with the presence of multiple components. However, it is noteworthy that these multiple peaks fall within the temperature range of BW, suggesting comparable thermal behaviour between the two materials.

The waxes exhibit similar thermal characteristics, as evidenced by their overlapping DP temperatures (61–70°C) and melting point ranges (64–70°C) in Table 2. The same range of DP temperatures suggests comparable performance and similar DP in finished products [47, 48].

The distinct profiles of their cooling and heating curves hint at potential implications for their application, such as solid anhydrous formulations like lipsticks and lip balms, where high MP waxes are crucial for achieving a well‐structured formula [27].

### Microscope study of oil–wax crosslinking

The next study was carried out to understand the interactions between oil and wax at a microscopic level.

Several studies have been performed on natural waxes, such as BW, with different oils. These studies showed that crystallization behaviour and physical properties of different natural waxes strongly depend on the type of wax and the type of oil used. The wax concentration also affects the physical properties of the material [49].

**FIGURE 2:**
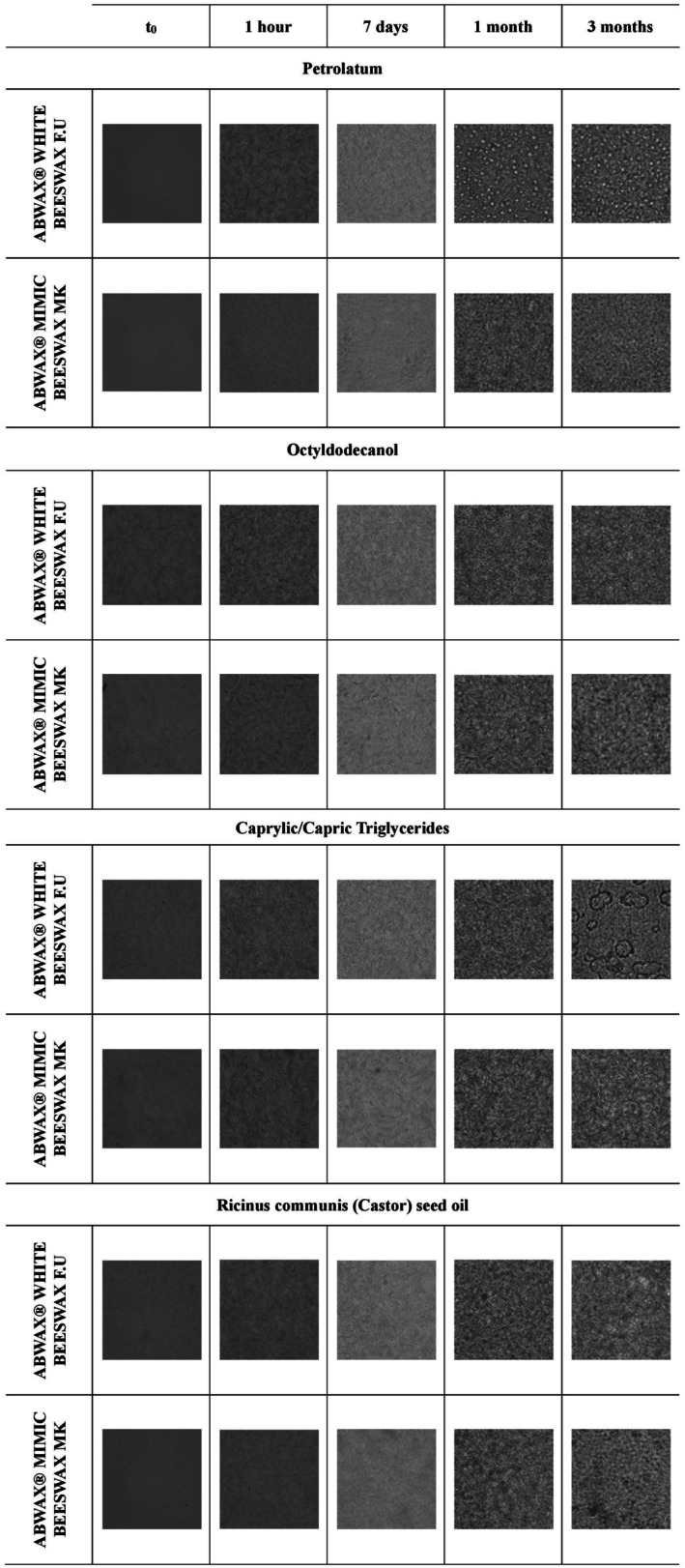
Microscope picture of oil–wax binary system (95% of oil and 5% of wax) crosslinking. Images were taken using an Olympus IX81 microscope (Olympus LS, Tokyo, Japan) equipped with an Optika C‐P20Cm digital camera (Optica Italia, Ponteranica, Italy). The microscope was set to a magnification of 40×, and an image of each sample was captured at *t*_0_ (immediately after melting the wax). The samples were then analysed again after 1 h, 7 days, 1 month and 3 months.

The microscope pictures in Figure 2 show a binary system made with 5% of ABWAX WHITE BEESWAX F.U. or ABWAX MIMIC BEESWAX MK and 95% of different oil over time. The percentage of BW and vBWA was chosen based on average usage in finished cosmetic products [19].

Petrolatum‐based synergies present comparative consistency between 1% and 3% wax concentrations (Figures S1 and S2). However, as the wax content increased to 5%, distinct morphological variations emerged. At this concentration, the BW sample exhibited a heterogeneous microstructure, characterized by the formation of well‐defined “spherical entities” over a one‐month observation period. This phenomenon was notably absent in the vBWA‐based synergies.

A kinetic assessment of the samples' behaviour revealed an absence of crystallization at time zero (*t*_0_). However, as time progressed, a dynamic transformation in morphology became evident. The BW and its alternative exhibited distinct behaviour patterns when synergized with petrolatum.

Although not widely used in modern cosmetics, to achieve a comprehensive investigation of oil–wax interactions, petrolatum was included in the study design, being an apolar hydrocarbon‐based oil.

The results with Octyldodecanol revealed that both BW and its alternative exhibited similar behaviours across all analysed concentrations: 1%, 3%, 5% and 10% of waxes, from both a kinetic and thermodynamic perspective (Figures S3 and S4 for 1%, 3% and 10% of BW and vBWA, Figure 2 for 5% of BW and vBWA). This suggests that the network formation process between the wax and the oil is highly dependent on the specific oil utilized. These findings highlight the importance of oil selection when formulating cosmetics involving waxes. The choice of oil can significantly influence the physical properties of the final product, such as viscosity, texture and stability.

Both Caprylic/Capric Triglycerides and Ricinus communis (Castor) seed oil exhibited similar behaviours at all investigated BW and vBWA concentration, 1%,3%, 5% and 10%, from both kinetic and thermodynamic perspectives (Caprylic/Capric Triglycerides: 1%, 3% and 10% of BW and vBWA Figures S5 and S6, Figure 2 for 5% of BW and vBWA; Ricinus communis (Castor) seed oil: 1%, 3% and 10% of BW and vBWA Figures S7 and S8, Figure 2 for 5% of BW and vBWA). Microscopic investigation of wax–oil synergies revealed a correlation of network formation on oil polarity. For polar oils (Octyldodecanol, Caprylic/Capric Triglycerides and Ricinus communis (Castor) seed), a consistent network arrangement was observed across all oil concentrations, suggesting a strong affinity between polar oils and waxes. In contrast, non‐polar oils exhibited variations in network formation beyond the 5% of wax concentration, highlighting the complex interplay between wax and oil polarities. The network formation patterns of non‐polar oil synergies exhibited time‐dependent changes. Over a period of approximately 1 month, the network structure of these synergies became more pronounced, suggesting a gradual reorganization of the wax and oil molecules. A previous study by Winkler‐Moser investigated the influence of BW concentration and oil type on network morphology. They observed that the network structure depends on both factors. At 2.5% BW concentration, olive oil exhibited a combination of small spherulites and needle‐edged crystals, while other oils formed only needle‐edged crystals. Interestingly, our findings align with theirs, as petrolatum displayed larger spherical agglomerates alongside needle‐edged crystals at 5% BW [39].

The present study delves into the relationship between wax type, oil polarity, network formation and time‐dependent changes, highlighting the crucial role of oil selection and wax concentration in determining the long‐term stability and performance of wax‐based formulations. These findings bring attention to the importance of considering both wax concentration and oil type when designing wax‐based formulations with desired network characteristics.

In line with the typical BW concentration range of 5%–6% in cosmetic products and the results obtained during this test, the following tests will be carried out with a percentage of use of BW and its alternative up to 5% and in synergy with a structuring wax, the SFW.

### Oil–wax synergies study

The crystallization behaviour of waxes significantly influences product performance [50]. The selection and blending of waxes with different MP is crucial for achieving the desired product characteristics [19]. High MP waxes provide structural integrity and shape retention, while low‐MP waxes contribute to product spreadability and emoliency.

The next step was to carry out a study on the hardness of the oil–wax systems using SFW as a structuring agent, as it exhibits the most promising gelling results compared to other structuring agents [51, 52].

BW provides a glossy finish due to its glazing properties, helps harden the stick for easy application and offers moisturizing benefits for dry lips [19].

Penetration value of the oil–wax blend provides information about the plasticity of the lipstick. A high value means deeper penetration in the sample, indicating a softer stick that releases more oil upon application. This analysis is crucial as hardness directly impacts performance [53].

DP analysis can also help to predict the texture of the final product. A higher difference between body temperature and DP results in a less smooth application, while a DP temperature close to body temperature suggests a creamier texture that releases more oil.

To evaluate the performance of ABWAX® WHITE BEESWAX F.U. and ABWAX® MIMIC BEESWAX MK, a sensory analysis was conducted alongside an instrumental analysis. The sensory panel assessed creaminess on a scale of 1 (not creamy) to 10 (very creamy) and spreadability using a rating system: 1–3 for low spreadability, 4–6 for medium spreadability and 7–10 for high spreadability.

**FIGURE 3:**
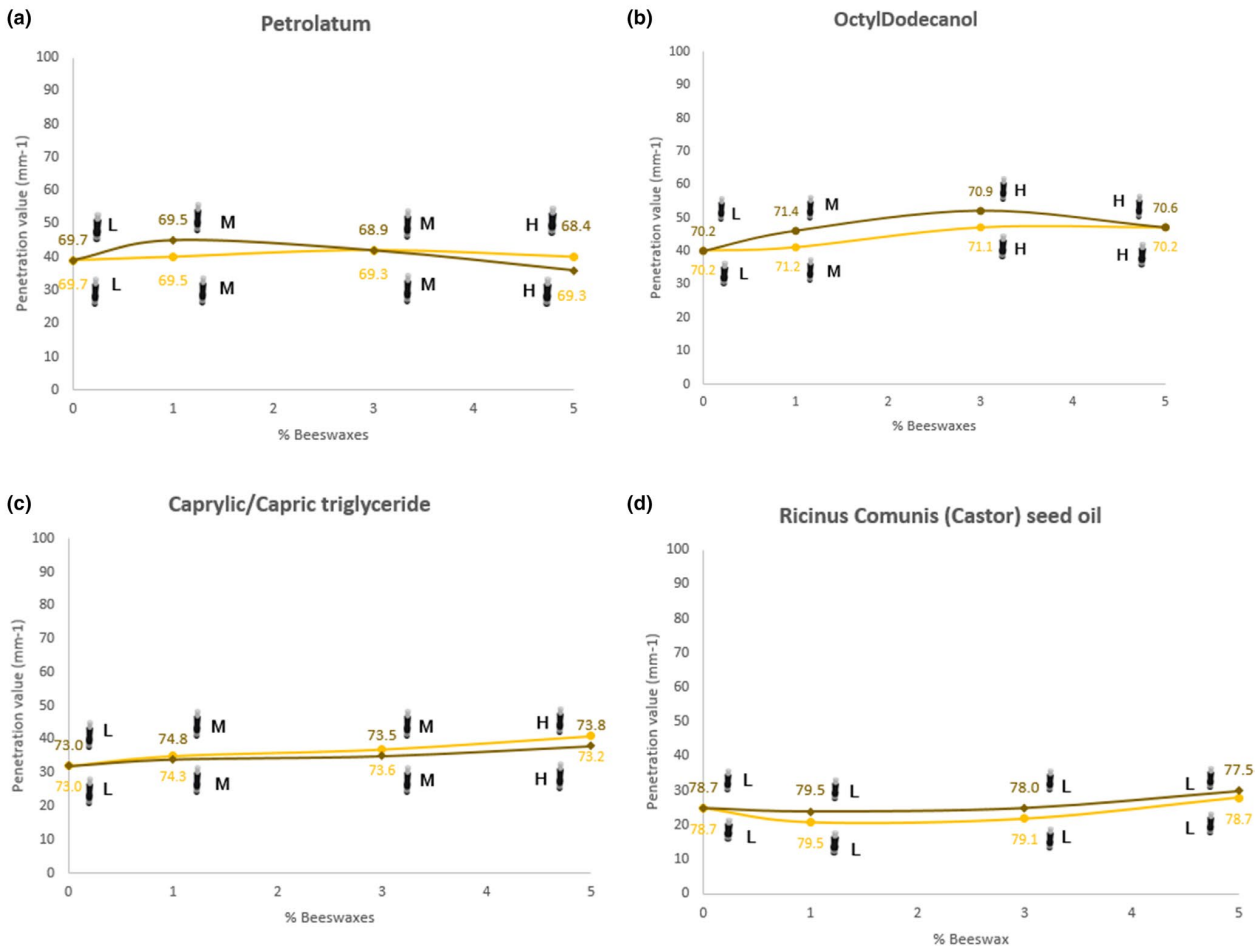
Penetration curves of the samples made of 25% BW‐SFW or vBWA‐SFW (yellow: ABWAX® WHITE BEESWAX F.U., brown: ABWAX® MIMIC BEESWAX MK) and 75% of Petrolatum (a), Octyldodecanol (b), Caprylic/Capric triglyceride (c) or Ricinus Communis (Castor) seed oil (d). To indicate whether a stick was formed at a specific BW concentration, an image of a stick is added to the curves. Spreadability is categorized as L (low), M (medium) or H (high).

In Figure 3a, Ternary synergies with Petrolatum revealed that the two waxes (BW and vBWA) exhibited similar behaviors across all tested percentages. At low percentages of both, the resulting sticks were classified as control with low spreadability. However, as the percentage of BW and vBWA increased, spreadability also improved, leading to sticks with high spreadability.

Figure 3b demonstrates similar performance of the two waxes with Octyldodecanol. As the content of BW and vBWA increases from 0% to 5%, the spreadability rating improves. Sticks containing no BWs exhibit a low spreadability. With the introduction of 1% BW and vBWA, the rating increases to a medium spreadability. Further increasing the BW or vBWA concentration to 3% and 5% results in consistently high spreadability ratings (8 for both).

Figure 3c shows very similar penetration values and performance between BW and vBWA with Caprylic/Capric triglyceride. As the BW content increases from 0% to 5%, the spreadability rating improves.

Figure 3d displays the penetration profile using Ricinus Communis (Castor) seed oil. As the concentration of BWs increases (from 0% to 5%) the penetration value and the DP value remain relatively constant. Furthermore, the values of penetration and DP were similar for ABWAX® WHITE BEESWAX F.U. and ABWAX® MIMIC BEESWAX MK, exhibiting comparable structuring properties. Sensory analysis rated all the samples as control sticks (two in both cases) with low spreadability (two for ABWAX® WHITE BEESWAX F.U. and three for ABWAX® MIMIC BEESWAX MK).

Our study highlights that waxes exhibit different structuring behaviours depending on the oil's polarity and viscosity. Penetration depth increases with increasing oil polarity. This phenomenon is attributed to the formation of smaller and more compact crystalline structures within the wax matrix when interacting with highly polar oils [54, 55].

Consequently, achieving the desired stick hardness can be controlled through a careful selection of wax concentration and the choice of oils based on their specific polarity and viscosity [45].

The addition of ABWAX® WHITE BEESWAX F.U. and ABWAX® MIMIC BEESWAX MK increases the spreadability of the sticks made with hydrocarbons, fatty alcohols and low molecular weight oils. The effect is less pronounced with high molecular weight oils. This observation can be attributed to the high viscosity of Ricinus Communis (Castor) seed oil, which limits its ability to spread.

All stick products were successfully extruded, and there were no significant differences in the values obtained for penetration, DP, spreadability and creaminess between ABWAX® WHITE BEESWAX F.U. and ABWAX® MIMIC BEESWAX MK.

The sample was then analysed using an optical microscope to understand the interactions between oils and waxes at a microscopic level.

**FIGURE 4:**
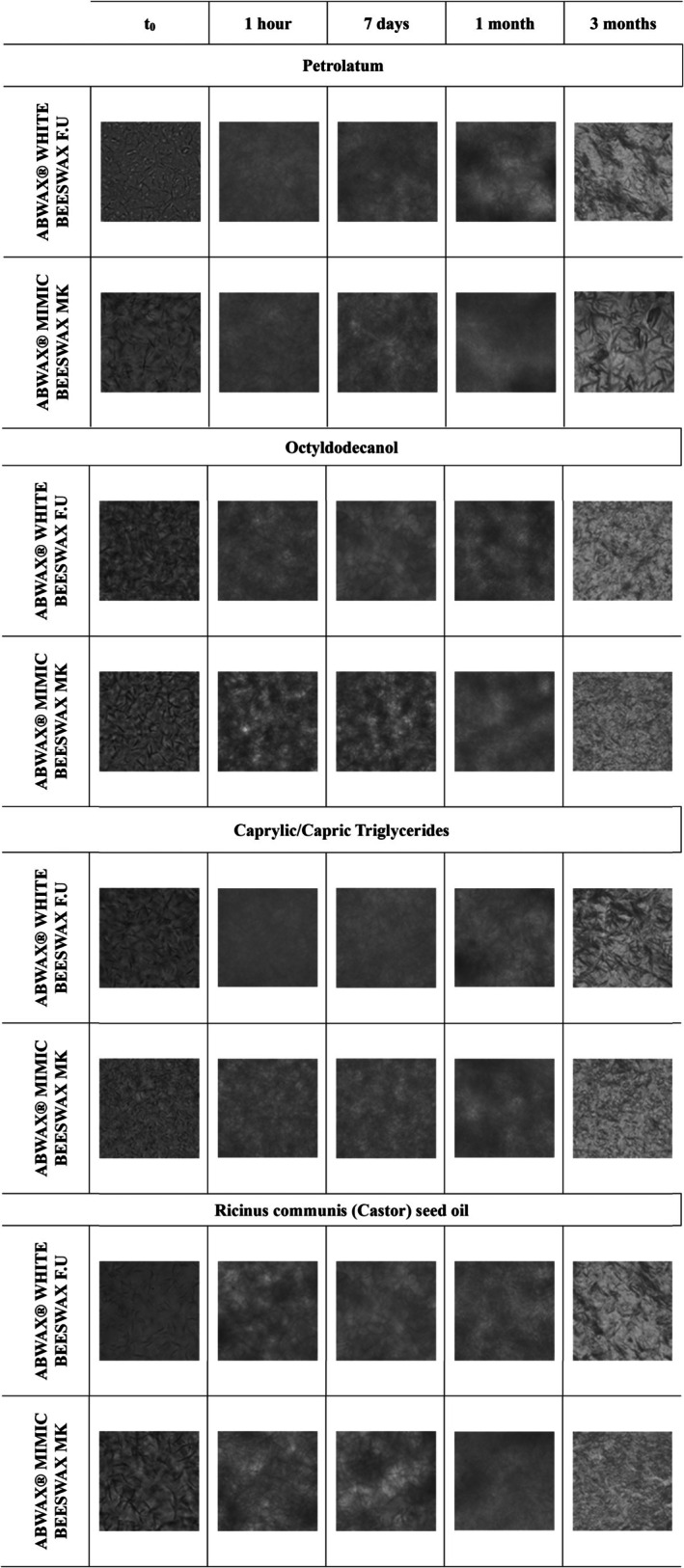
Microscope picture of oil–wax crosslinking with the combination of 25% of 5:95 wax: SFW and 75% of oil. Images were taken using an Olympus IX81 microscope (Olympus LS, Tokyo, Japan) equipped with an Optika C‐P20Cm digital camera (Optica Italia, Ponteranica, Italy). The microscope was set to a magnification of 40×, and an image of each sample was captured at *t*_0_ (immediately after melting the wax). The samples were then analysed again after 1 h, 7 days, 1 and 3 months.

The microscope pictures in Figure 4 show a ternary system made with 25% synergies of BW‐SFW or vBWA‐SFW and 75% of different oils over time.

Microscopic observations of SFW–oil systems in previous studies reported distinct crystal morphologies dependent on the specific oil used. These morphologies typically included needle‐like crystals and large, flat crystals known as platelets [39, 50]. Instead, in our ternary systems formulated with SFW and different oils where BW/vBWA were added, the crystalline arrangements still contain needle‐like crystals, but these are composed of smaller crystals forming a more homogeneous structure, different from what has already been reported by Martini and Winkler‐Moser.

This singular behaviourmay be attributable to the complex interactions that are generated between SFW and BW/vBWA.

*T*_0_ images of Petrolatum‐based ternary synergies reveal dark patches and striated fragments, which become more pronounced as the BW and vBWA content increases. Over time, these structures become less visible, and the samples become more homogeneous. ABWAX® WHITE BEESWAX F.U. exhibits well‐defined “spherical entities” at 5% *t*_0_, which were previously observed in the study of petrolatum‐BW synergies (95:5). These structures disappear over time, which were previously observed in the study of petrolatum‐BW synergies (95:5). These structures disappear over time.

The *t*_0_ images of the ternary system made of 25% BW‐SFW and vBWA‐SFW with 75% of octyldodecanol or caprylic/capric triglyceride reveal dark patches and striated fragments, which become more pronounced as the BWs content increases; these structures disappear over time.

The ternary system made with 25% synergies of BWs‐SFW and 75% of Ricinus Communis (Castor) seed oil reveals the same dark patches at *t*_0_ as the other oils. The images of ABWAX MIMIC BEESWAX MK show slightly more structures at *t*_0_ and t3M than BW.

### Development of finished products

This phase of the study focused on evaluating the compatibility of pigments and coated lakes with the oil–waxes synergies that demonstrated the best stability and thermal properties [53]. This study aimed to formulate a lipstick that delivered on sensory and aesthetic aspects, bringing the research to finished cosmetic formulation. The formula was developed, incorporating pigments and lakes with selected oils based on their viscosity and polarity. The resulting lipsticks (detailed in Table 3) were evaluated by a panel of 25 participants who assessed their application feel. Specific focus was placed on smoothness, control during application and overall comfort.

**TABLE 3:** Percentages of all the ingredients used for each finished product.

INCI	%
ABWAX® WHITE BEESWAX F.U./ABWAX® MIMIC BEESWAX MK	4.80
Helianthus Annuus Seed Cera	9.60
Ricinus communis (Castor) seed oil	28.20
Ethylhexyl Pelargonate	21.40
Triolein, Glyceryl Dioleate	21.00
CI 77491, C8‐12 Acid Triglyceride	10.00
Mica, CI 77891, Tin oxide	5.00

**FIGURE 5:**
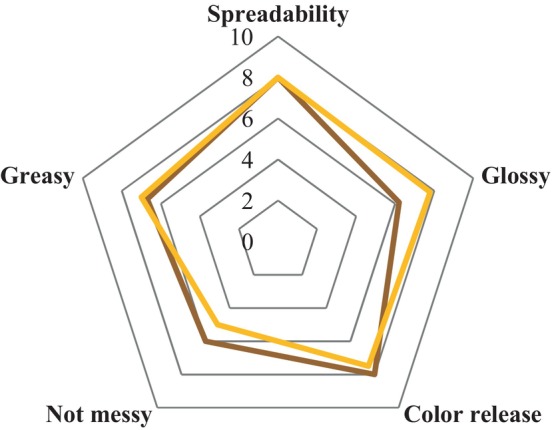
Comparison between Table 3 lipstick made with ABWAX® WHITE BEESWAX F.U. (Yellow) or ABWAX® MIMIC BEESWAX MK (Brown).

Figure 5 compares the finished lipstick from Table 3 made with ABWAX® WHITE BEESWAX F.U. and the one made with ABWAX® MIMIC BEESWAX MK.

The performances of the two sticks are similar in most parameters, including spreadability and greasiness. However, the ABWAX® WHITE BEESWAX F.U. formula displayed a slightly glossier finish, while the ABWAX® MIMIC BEESWAX MK formula received higher marks for application control and colour release from the panel.

Panellists described the ABWAX® MIMIC BEESWAX MK lipstick as feeling more manageable during application, while the ABWAX® WHITE BEESWAX F.U. lipstick, although glossier, felt too smooth and potentially difficult to apply precisely.

These findings suggest that in lipstick formulations, even if made of just a few ingredients, ABWAX® MIMIC BEESWAX MK can effectively replace ABWAX® WHITE BEESWAX F.U. in a 1:1 ratio without compromising performance, as evidenced by the panel test results. Notably, the ABWAX® MIMIC BEESWAX MK formulation also appears to offer superior wear comfort compared to the benchmark. These results highlight the potential of ABWAX® MIMIC BEESWAX MK as an alternative in lipstick formulations.

## CONCLUSION

This study explores a potential vegan alternative to traditional beeswax in cosmetic stick formulations. The analysis of the two raw materials revealed that ABWAX® WHITE BEESWAX F.U. and ABWAX® MIMIC BEESWAX MK exhibit comparable thermal and chemical characteristics.

Stick formulations made with oil of different nature at concentrations below 10% yielded encouraging results at both macro and micro levels.

Stick formulations are made with a total wax content of 25% and individual waxes are typically used at a concentration of 5% [51, 52]. The study found that the sticks formulated with a fixed wax (SFW), the two alternative waxes, and oils of varying nature demonstrated comparable performance in terms of texture, spreadability, thermal properties and hardness.

A case study of a finished product evaluated by 25 panellists revealed no significant differences between the vegan alternative and beeswax. This outcome highlights the potential of ABWAX® MIMIC BEESWAX MK as a viable alternative to beeswax in vegan cosmetic stick formulations.

The successful replacement of beeswax with ABWAX® MIMIC BEESWAX MK opens up possibilities for creating vegan and sustainable cosmetic formulations without compromising product performance or consumer satisfaction.

## CONFLICT OF INTEREST STATEMENT

The authors declare that they have no known competing financial interests or personal relationships that could have appeared to influence the work reported in this paper.

## Supporting information


Data S1
