# Supplementary material for: Formulation and evaluation of alternative to beeswax for vegan lipsticks
Source: Int J Cosmet Sci. 2025 Mar 12;47(4):626–38. doi: 10.1111/ics.13060 (PMC12319492; doi:10.1111/ics.13060)
Supplement: Supplementary file 1 — Data S1 [file ICS-47-626-s001.docx]

Supporting information for

**Formulation and Evaluation of Alternative to Beeswax for Vegan Make-Up**

*Manuela Loiacono^1^, Luigi Padovano^1^, Miryam Chiara Malacarne^2^, Simone Conti^1^, Enrico Caruso^2*^*

^1^ Brasca Industrial s.r.l. Via Nerviano 31, 20045 Lainate (MI), Italy.

^2^ Department of Biotechnology and Life Sciences (DBSV). University of Insubria, Via J.H. Dunant 3, 21100 Varese (VA), Italy.

*To whom correspondence should be addressed. Phone: +39-0331-421544. E-mail: enrico.caruso@uninsubria.it

# Microscope pictures of oil-wax crosslinking with the combination of 1:99, 3:97, 5:95 and 10:90 Wax:Oil. Images were taken using an Olympus IX81 microscope (Olympus LS, Tokyo, Japan) equipped with an Optika C-P20Cm digital camera (Optica Italia, Ponteranica, Italy). The microscope was set to a magnification of 40X, and an image of each sample was captured at t_0_ (immediately after melting the wax). The samples were then analysed again after 1 hour, 7 days, 1 month, and 3 months.

# **Figure S1.** Microscope picture of oil-wax crosslinking with the combination of 1:99, 3:97, 5:95 and 10:90 ABWAX WHITE BEESWAX F.U.: Petrolatum.

|  | **t_0_** | **1 hour** | **7 days** | **1 month** | **3 months** |
| --- | --- | --- | --- | --- | --- |
| **1%** | 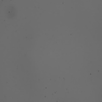 | 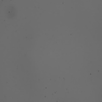 | 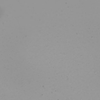 | 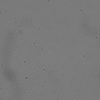 | 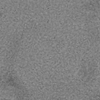 |
| **3%** | 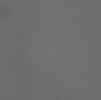 | 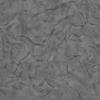 | 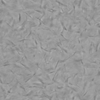 | 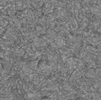 | 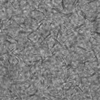 |
| **5%** | 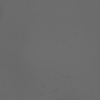 | 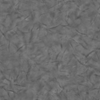 | 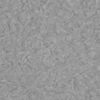 | 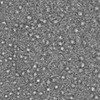 | 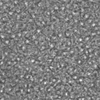 |
| **10%** | 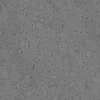 | 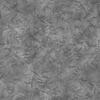 | 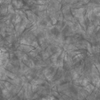 | 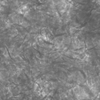 | 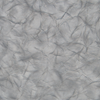 |

# **Figure S2.** Microscope picture of oil-wax crosslinking with the combination of 1:99, 3:97, 5:95 and 10:90 ABWAX MIMIC BEESWAX MK: Petrolatum.

|  | **t_0_** | **1 hour** | **7 days** | **1 month** | **3 months** |
| --- | --- | --- | --- | --- | --- |
| **1%** | 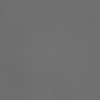 | 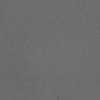 | 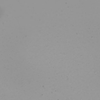 | 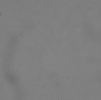 | 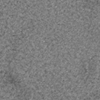 |
| **3%** | 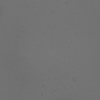 | 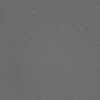 | 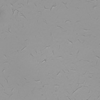 | 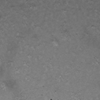 | 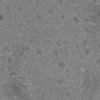 |
| **5%** | 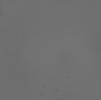 | 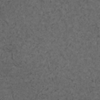 | 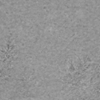 | 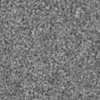 | 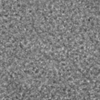 |
| **10%** | 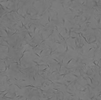 | 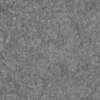 | 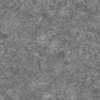 | 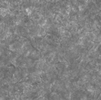 | 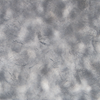 |

# **Figure S3.** Microscope picture of oil-wax crosslinking with the combination of 1:99, 3:97, 5:95 and 10:90 ABWAX WHITE BEESWAX F.U.: Octyldodecanol.

|  | **t_0_** | **1 hour** | **7 days** | **1 month** | **3 months** |
| --- | --- | --- | --- | --- | --- |
| **1%** | 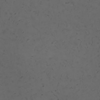 | 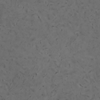 | 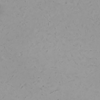 | 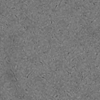 | 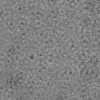 |
| **3%** | 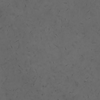 | 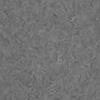 | 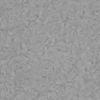 | 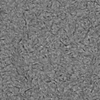 | 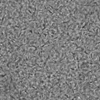 |
| **5%** | 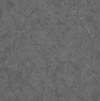 | 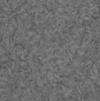 | 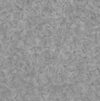 | 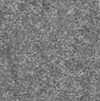 | 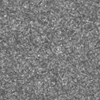 |
| **10%** | 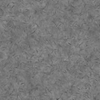 | 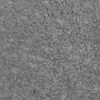 | 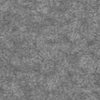 | 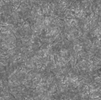 | 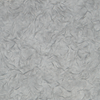 |

**Figure S4.** Microscope picture of oil-wax crosslinking with the combination of 1:99, 3:97, 5:95 and 10:90 ABWAX MIMIC BEESWAX MK: Octyldodecanol.

|  | **t_0_** | **1 hour** | **7 days** | **1 month** | **3 months** |
| --- | --- | --- | --- | --- | --- |
| **1%** | 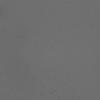 | 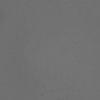 | 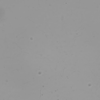 | 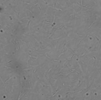 | 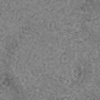 |
| **3%** | 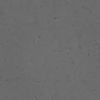 | 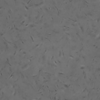 | 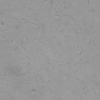 | 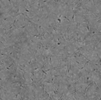 | 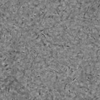 |
| **5%** | 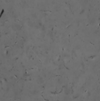 | 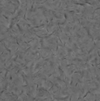 | 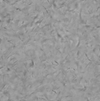 | 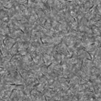 | 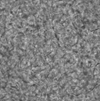 |
| **10%** | 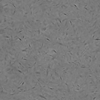 | 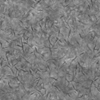 | 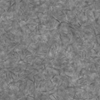 | 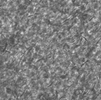 | 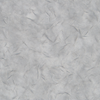 |

**Figure S5.** Microscope picture of oil-wax crosslinking with the combination of 1:99, 3:97, 5:95 and 10:90 ABWAX WHITE BEESWAX F.U.: Caprylic/Capric Triglycerides.

|  | **t_0_** | **1 hour** | **7 days** | **1 month** | **3 months** |
| --- | --- | --- | --- | --- | --- |
| **1%** | 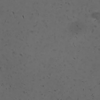 | 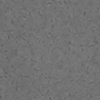 | 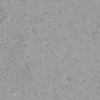 | 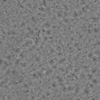 | 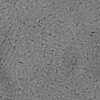 |
| **3%** | 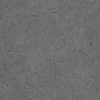 | 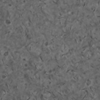 | 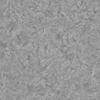 | 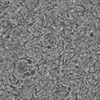 | 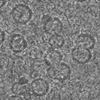 |
| **5%** | 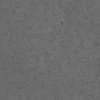 | 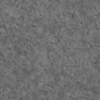 | 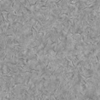 | 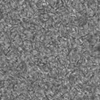 | 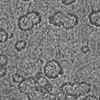 |
| **10%** | 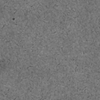 | 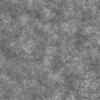 | 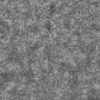 | 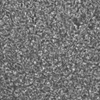 | 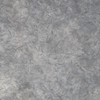 |

# **Figure S6.** Microscope picture of oil-wax crosslinking with the combination of 1:99, 3:97, 5:95 and 10:90 ABWAX MIMIC BEESWAX MK: Caprylic/Capric Triglycerides.

|  | **t_0_** | **1 hour** | **7 days** | **1 month** | **3 months** |
| --- | --- | --- | --- | --- | --- |
| **1%** |  |  |  |  |  |
| **3%** |  |  |  |  |  |
| **5%** |  |  |  |  |  |
| **10%** |  |  |  |  |  |

# **Figure S7.** Microscope picture of oil-wax crosslinking with the combination of 1:99, 3:97, 5:95 and 10:90 ABWAX WHITE BEESWAX F.U.: Ricinus communis seed oil.

|  | **t_0_** | **1 hour** | **7 days** | **1 month** | **3 months** |
| --- | --- | --- | --- | --- | --- |
| **1%** |  |  |  |  |  |
| **3%** |  |  |  |  |  |
| **5%** |  |  |  |  |  |
| **10%** |  |  |  |  |  |

# **Figure S8.** Microscope picture of oil-wax crosslinking with the combination of 1:99, 3:97, 5:95 and 10:90 ABWAX MIMIC BEESWAX MK: Ricinus communis seed oil.

|  | **t_0_** | **1 hour** | **7 days** | **1 month** | **3 months** |
| --- | --- | --- | --- | --- | --- |
| **1%** |  |  |  |  |  |
| **3%** |  |  |  |  |  |
| **5%** |  |  |  |  |  |
| **10%** |  |  |  |  |  |

# Microscope pictures of oil-wax crosslinking with the combination of 25% of synergies 1:24, 3:22, 5:20 Wax:SFW and 75% of Oil. Images were taken using an Olympus IX81 microscope (Olympus LS, Tokyo, Japan) equipped with an Optika C-P20Cm digital camera (Optica Italia, Ponteranica, Italy). The microscope was set to a magnification of 40X, and an image of each sample was captured at t_0_ (immediately after melting the wax). The samples were then analysed again after 1 hour, 7 days, 1 month, and 3 months.

# **Figure S9.** Microscope picture of oil-wax crosslinking with the combination of 25% of synergies 1:24, 3:22, 5:20 ABWAX WHITE BEESWAX F.U.: SFW and 75% of Petrolatum.

|  | **t_0_** | **1 hour** | **7 days** | **1 month** | **3 months** |
| --- | --- | --- | --- | --- | --- |
| **1%** |  |  |  |  |  |
| **3%** |  |  |  |  |  |
| **5%** |  |  |  |  |  |

# **Figure S10.** Microscope picture of oil-wax crosslinking with the combination of 25% of synergies 1:24, 3:22, 5:20 ABWAX MIMIC BEESWAX MK.: SFW and 75% of Petrolatum.

|  | **t_0_** | **1 hour** | **7 days** | **1 month** | **3 months** |
| --- | --- | --- | --- | --- | --- |
| **1%** |  |  |  |  |  |
| **3%** |  |  |  |  |  |
| **5%** |  |  |  |  |  |

# **Figure S11.** Microscope picture of oil-wax crosslinking with the combination of 25% of synergies 1:24, 3:22, 5:20 ABWAX WHITE BEESWAX F.U.: SFW and 75% of Octyldodecanol.

|  | **t_0_** | **1 hour** | **7 days** | **1 month** | **3 months** |
| --- | --- | --- | --- | --- | --- |
| **1%** |  |  |  |  |  |
| **3%** |  |  |  |  |  |
| **5%** |  |  |  |  |  |

# **Figure S12.** Microscope picture of oil-wax crosslinking with the combination of 25% of synergies 1:24, 3:22, 5:20 ABWAX MIMIC BEESWAX MK.: SFW and 75% of Octyldodecanol.

|  | **t_0_** | **1 hour** | **7 days** | **1 month** | **3 months** |
| --- | --- | --- | --- | --- | --- |
| **1%** |  |  |  |  |  |
| **3%** |  |  |  |  |  |
| **5%** |  |  |  |  |  |

# **Figure S13.** Microscope picture of oil-wax crosslinking with the combination of 25% of synergies 1:24, 3:22, 5:20 ABWAX WHITE BEESWAX F.U.: SFW and 75% of Caprylic/Capric triglyceride.

|  | **t_0_** | **1 hour** | **7 days** | **1 month** | **3 months** |
| --- | --- | --- | --- | --- | --- |
| **1%** |  |  |  |  |  |
| **3%** |  |  |  |  |  |
| **5%** |  |  |  |  |  |

# **Figure S14.** Microscope picture of oil-wax crosslinking with the combination of 25% of synergies 1:24, 3:22, 5:20 ABWAX MIMIC BEESWAX MK.: SFW and 75% of Caprylic/Capric triglyceride.

|  | **t_0_** | **1 hour** | **7 days** | **1 month** | **3 months** |
| --- | --- | --- | --- | --- | --- |
| **1%** |  |  |  |  |  |
| **3%** |  |  |  |  |  |
| **5%** |  |  |  |  |  |

# **Figure S15.** Microscope picture of oil-wax crosslinking with the combination of 25% of synergies 1:24, 3:22, 5:20 ABWAX WHITE BEESWAX F.U.: SFW and 75% of Ricinus Communis (Castor) seed oil.

|  | **t_0_** | **1 hour** | **7 days** | **1 month** | **3 months** |
| --- | --- | --- | --- | --- | --- |
| **1%** |  |  |  |  |  |
| **3%** |  |  |  |  |  |
| **5%** |  |  |  |  |  |

# **Figure S16.** Microscope picture of oil-wax crosslinking with the combination of 25% of synergies 1:24, 3:22, 5:20 ABWAX MIMIC BEESWAX MK.: SFW and 75% of Ricinus Communis (Castor) seed oil.

|  | **t_0_** | **1 hour** | **7 days** | **1 month** | **3 months** |
| --- | --- | --- | --- | --- | --- |
| **1%** |  |  |  |  |  |
| **3%** |  |  |  |  |  |
| **5%** |  |  |  |  |  |
